# Functional MRI Correlates of Carbon Dioxide Chemosensing in Persons With Epilepsy

**DOI:** 10.3389/fneur.2022.896204

**Published:** 2022-07-07

**Authors:** Johnson P. Hampson, Nuria Lacuey, MR Sandhya Rani, Jaison S. Hampson, Kristina A. Simeone, Timothy A. Simeone, Ponnada A. Narayana, Louis Lemieux, Samden D. Lhatoo

**Affiliations:** ^1^Department of Neurology, McGovern Medical School, University of Texas Health Science Center at Houston, Houston, TX, United States; ^2^Department of Pharmacology and Neuroscience, Creighton University School of Medicine, Omaha, NE, United States; ^3^Department of Diagnostic and Interventional Imaging, McGovern Medical School, University of Texas Health Science Center at Houston, Houston, TX, United States; ^4^Department of Clinical and Experimental Epilepsy, University College London (UCL) Institute of Neurology, National Hospital for Neurology and Neurosurgery, London, United Kingdom

**Keywords:** sudden unexplained death in epilepsy (SUDEP), epilepsy, functional MRI (fMRI), functional connectivity, hypercapnia

## Abstract

**Objectives:**

Sudden unexpected death in epilepsy (SUDEP) is a catastrophic epilepsy outcome for which there are no reliable premortem imaging biomarkers of risk. Percival respiratory depression is seen in monitored SUDEP and near SUDEP cases, and abnormal chemosensing of raised blood carbon dioxide (CO_2_) is thought to contribute. Damage to brainstem respiratory control and chemosensing structures has been demonstrated in structural imaging and neuropathological studies of SUDEP. We hypothesized that functional MRI (fMRI) correlates of abnormal chemosensing are detectable in brainstems of persons with epilepsy (PWE) and are different from healthy controls (HC).

**Methods:**

We analyzed fMRI BOLD activation and brain connectivity in 10 PWE and 10 age- and sex-matched HCs during precisely metered iso-oxic, hypercapnic breathing challenges. Segmented brainstem responses were of particular interest, along with characterization of functional connectivity metrics between these structures. Regional BOLD activations during hypercapnic challenges were convolved with hemodynamic responses, and the resulting activation maps were passed on to group-level analyses. For the functional connectivity analysis, significant clusters from BOLD results were used as seeds. Each individual seed time-series activation map was extracted for bivariate correlation coefficient analyses to study changes in brain connectivity between PWE and HCs.

**Results:**

(1) Greater brainstem BOLD activations in PWE were observed compared to HC during hypercapnic challenges in several structures with respiratory/chemosensing properties. Group comparison between PWE vs. HC showed significantly greater activation in the dorsal raphe among PWE (*p* < 0.05) compared to HCs. (2) PWE had significantly greater seed-seed connectivity and recruited more structures during hypercapnia compared to HC.

**Significance:**

The results of this study show that BOLD responses to hypercapnia in human brainstem are detectable and different in PWE compared to HC. Increased dorsal raphe BOLD activation in PWE and increased seed-seed connectivity between brainstem and adjacent subcortical areas may indicate abnormal chemosensing in these individuals. Imaging investigation of brainstem respiratory centers involved in respiratory regulation in PWE is an important step toward identifying suspected dysfunction of brainstem breathing control that culminates in SUDEP and deserve further study as potential imaging SUDEP biomarkers.

## Key Points

- Functional MRI can detect abnormal chemosensing in brainstem structures.
- Dorsal raphe a region rich in serotonergic neurons plays a major role in responding to changes in carbon dioxide chemosensing.
- Functional MRI can serve as a biomarker for SUDEP risk among patients with epilepsy.

## Introduction

Sudden unexpected death in epilepsy (SUDEP) affects up to 1% of persons with medically refractory epilepsy (1, 2). It is a catastrophic outcome of epilepsy, most common in young patients who are otherwise healthy. Despite its major impact on life expectancy in refractory epilepsy, no reliable SUDEP biomarkers currently exist and thus represent a critical unmet need for targeted SUDEP intervention.

Observational studies point to the failure of recovery from peri-ictal respiratory depression as a main concern (3, 4). Structural and/or functional compromise of brainstem breathing control networks appears likely. Structural brain MRI of subjects who subsequently succumbed to SUDEP typically indicates volume loss in key autonomic and breathing control sites (periaqueductal gray, raphe nuclei, and medial posterior thalamus) (5, 6). In support of these observations, a series of SUDEP postmortem cases have shown significant reductions in pacemaker-like somatostatin and neurokinin-1 receptor-positive neurons in the ventrolateral medulla, suggesting that both structural and functional brainstem abnormalities exist in patients with SUDEP (7). These findings indicate epilepsy-mediated pathology in brainstem respiratory neuronal groups that promote vulnerability to SUDEP.

Severe and prolonged increases in end-tidal CO_2_ (ETCO_2_) occur with seizures(8). In a recent study of PWE, decreased interictal hypercapnic ventilatory responses (HCVR) to CO_2_ were correlated with higher and more prolonged peri-ictal hypercapnia (9). HCVRs seem to vary greatly in PWE, and thus, alternative measures of capturing brainstem dysfunction are attractive (9). One imaging study done in healthy human volunteers showed activation of the thalamus, the inferior ventral and dorsal rostral pons, and dorsal and lateral medulla in response to CO_2_ (10), indicating feasibility in PWE.

Premortem identification of compromised structural and functional respiratory control would allow targeted intervention and prevention of SUDEP risk. The primary objective of this work was to identify the activation of brainstem respiratory centers. We hypothesized that blood oxygenation level-dependent (BOLD) responses to increase CO_2_ (hypercapnic challenges) are observable in humans and are different in PWE compared to HC. This could potentially lead to the development of premortem risk markers for SUDEP.

## Methods

We investigated brain connectivity alterations in response to hypercapnic challenges in PWE and HC. We were particularly interested in segmenting brainstem responses to hypercapnic challenges and characterizing functional connectivity metrics of these structures.

### Participants

The study was approved by the University of Texas Health Sciences Center Houston Institutional Review Board, and participants provided written informed consent. Study patients were recruited from the Adult Epilepsy Monitoring Unit and University of Texas Health Epilepsy clinic. We included patients with a diagnosis of epilepsy, aged 18–60 years, who had not undergone resective brain surgery. Exclusion criteria were as follows: use of antidepressant medications, history of cardiac disease, respiratory disease, claustrophobia, metallic implants or devices (e.g., implantable cardioverter-defibrillator [ICD], pacemaker, embolic coils, aneurysm clips), or other material potentially hazardous in the MRI environment), bodyweight < 275 pounds (125 kg—scanner restrictions), stereoelectroencephalography (SEEG) implantation in the preceding 6 months, pregnancy, history of stroke, diagnosis of psychiatric disease, airway or chest deformities interfering with breathing, mechanical ventilatory or circulatory support, and renal failure.

Subjects were asked to refrain from consuming food or beverages with vasoactive effects, e.g., coffee, tea, and herbal remedies, at least 12 h prior to the fMRI scan. The participant's systolic, diastolic, and mean blood pressure and peripheral oxygen saturation (SpO_2_) were measured before and after the breathing challenge fMRI session. A multidimensional dyspnea profile (MDP) survey (11) was also completed by each participant at the end of the study to gauge overall unpleasantness from the breathing challenge.

### Functional MRI Data Acquisition and Analysis

Functional MRI scans were performed on a 3T scanner (Ingenia; Philips Medical Systems) using a 16-channel SENSE head coil. An anatomical overlay scan was acquired using a 3D T1 weighted high-resolution structural scan for normalization using the following parameters (TR/TE: 9.79/4.5 ms, FA = 6, FOV = 256 mm × 256 mm × 176 mm, matrix size 320 × 320 matrix with 220 slices and 0.80 mm in-plane resolution). Whole-brain blood oxygenation level-dependent (BOLD) functional scans were acquired using a T2^*^-weighted echo-planar sequence using the following parameters: TR/TE 2,400/30 ms, flip angle = 85°, FOV = 224 mm, 48 transverse slices, thickness 3.5 mm × 3.5 mm^2^ in-plane resolution. The fMRI protocol consisted of 346 volumes (duration: 13 min 50.4 s) covering the whole brain from the top of the cortex to the base of the brain stem.

Head motion was restricted using foam pads and a forehead strap. Comprehensive training and familiarization with the breathing challenge (dummy runs) were completed prior to MRI to reduce any anxiety with the challenge and to encourage completion of the task.

#### Breathing Challenge

For the fMRI breathing challenge, we used the well-established physiological research RespirAct™ (RA) device (12, 13). The RA device can deliver precise vasoactive stimuli (CO_2_) during MRI for whole-brain mapping of the cerebral blood flow responses. It controls alveolar ventilation, enabling targeted reproducible changes in arterial CO_2_ levels (PaCO_2_), such that CO_2_ targets set on RA are identical to measure arterial CO_2_ (12, 13). This enabled precise and reproducible “step” increases in CO_2_ up to 10 mmHg above resting levels in 1 to 2 breaths for the measurement of BOLD responses to CO_2_. Using this device, each participant underwent a 14-min block-design scan, consisting of 2-min blocks of breathing challenges interleaved with rest. The respiratory rate, end tidal CO_2_ (EtCO_2_), and end tidal O_2_ (EtO_2_) were continuously recorded throughout the scan. Gas supply to mask and breathing circuit was done through a programmable computer-controlled gas delivery system containing a non-rebreathing valve that directs blended gas to an inspiratory gas reservoir and patient. The breathing challenge protocol, previously established in cerebrovascular studies (12), involved two iso-oxic patterns of CO_2_ modulation: The first comprised square wave increases in EtCO_2_ by 10 mmHg from individual baseline value for 120 s, followed by 120 s of baseline. The second sequence was a slow ramp increase of EtCO_2_ by 15 mmHg from individual baseline for 120 s followed by baseline (120 s). Patients were administered precisely metered CO_2_ not exceeding pCO_2_ of 50 mmHg, with iso-oxygenation maintained at 110 mmHg at all times. Gas calibrations were performed using an EPA industry-standard calibration gas mixture before the start of each scan. Individual breath-by-breath inspired gas and flow concentrations were pre-calculated before the breathing challenge to establish individual's baseline levels and to set end-tidal gas concentration targets.

### Analyses

Data were pre-processed and analyzed using SPM software package version 12 (Statistical Parametric Mapping; Wellcome Department of Cognitive Neurology, London, United Kingdom), as well as the functional connectivity toolbox Conn (Cognitive and Affective Neuroscience Laboratory, Massachusetts Institute of Technology, Cambridge, USA) running under MATLAB 2016a (Mathworks, Sherborn, MA, USA). fMRI data were quality checked for excessive motion. Subject head motion was assessed by evaluating three translations and three rotations motions for each scan. Translational thresholds were set to ± 2 mm, whereas rotational thresholds were limited to ± 1°. Pre-processing steps included slice time correction, CompCor physiological noise correction (14), co-registration (realignment to the first image of the time series), normalization to MNI space (Montreal Neurological Institute), and smoothing with a Gaussian kernel of 6 mm full width at half maximum (FWHM) to compensate for small residual anatomic variations across participants.

#### BOLD Comparison During Breathing Challenge

**Figure 1:**
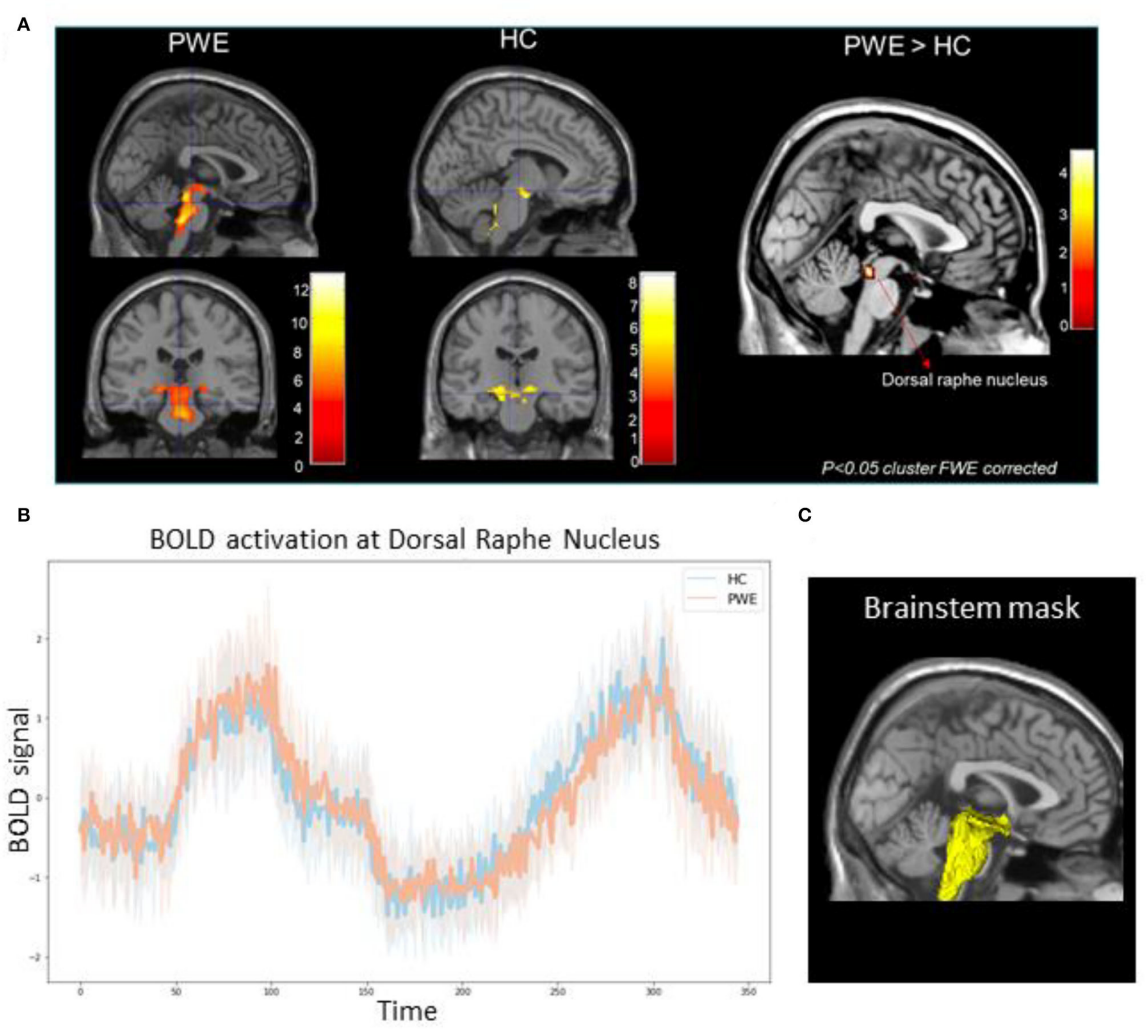
**(A)** Group-level brainstem BOLD activation differences among 10 patients (PWE) and 10 healthy controls (HC) during CO_2_ challenge showed greater activations among PWE at the dorsal raphe nucleus (p_FWE_ <0.05) compared to HCs. Color bars represent Z-score activation values. **(B)** Line plot represents mean BOLD activation time series extraction from all PWE (orange) and HCs (blue) at the dorsal raphe nucleus in response to the step and ramp challenge. Shaded hue areas represent corresponding individual confidence intervals. **(C)** 3D reconstruction of the explicit brainstem plus binary mask (yellow) used in the analysis overlaid over standard template T1 slice.

A first-level, individual subject analysis was performed using the general linear model implemented in SPM12. Motion parameters from motion correction re-alignment were modeled as regressors of no interest. Breathing challenge blocks (step + ramp response) were convolved with the canonical hemodynamic response function, and resulting activation maps were then passed up to group-level analyses comparing the differences in activation among the patient and healthy participant cohorts using age as a covariate of no interest. Using the available probabilistic brainstem segmentation atlas (15, 16), we created a custom explicit brainstem plus binary mask to study BOLD activation changes in the brainstem hypothalamus, and posterior thalamus structures during both the step and ramp breathing challenge (shown in Figure 1C). This mask served to spatially remove confounds from physiological noise found in areas adjacent to the brainstem. Second-order group analysis was performed to compare the breathing challenge-related BOLD changes between PWE and HC groups. The minimum threshold for all analysis results was set at *p*-value <0.05 family-wise-error (FWE) cluster-corrected significance (17).

#### Functional Connectivity During Breathing Challenge

We investigated the breathing challenge-related changes in brain connectivity in the patient groups and healthy controls. Seed regions were identified from the significant group comparison BOLD results from both PWE and HCs. To this effect, regions of interest corresponding to each significant cluster consisted of a sphere (3 mm radius) around the cluster's most significant voxel (shown in Table 1); larger activation clusters were further subdivided into sub-regions based on the location of peak voxels, resulting in a set of seed regions. Individual structural CSF and motion parameters were entered into the analysis as covariates of no interest. A band-pass filter (frequency window: 0.008–0.09 Hz) was applied to remove linear drifts and high-frequency noise from the data. For each subject, regional mean BOLD time series was estimated for each seed by averaging the time series of all voxels. The bivariate correlation coefficient was used to measure the level of linear association between the BOLD time series of every seed region pair (18). The resulting one-sample connectivity maps were compared among PWE and HC groups, using a multivariate statistic parametric (MVPA) omnibus test for cluster-level interference (19), adding age as a covariate of no interest (20). The resulting cluster maps were threshold at *p* < 0.05 FDR/FWE cluster correction for multiple comparisons.

**Table 1:** Demographic, cardiorespiratory, and multidimensional dyspnea profile data; group comparisons and changes.

	**PWE (*n* = 10)**	**HC (*n* = 10)**	***p*-value**
**Age, mean** **±SD**	35.9 ± 14.2	33.4 ± 7.3	*P* = 0.63
**Sex, N**	5 M, 5 F	5 M, 5 F	
**BMI, mean** **±SD**	22.7 ± 4.2	25.6 ± 6.1	*P* = 0.23
**Cardiovascular measurements Baseline, mean** **±SD**			
∘ Systolic	121.4 ± 16.4	127.3 ± 12.5	*P* = 0.91
∘ Diastolic	70.2 ± 8.9	75.7 ± 7.1	*P* = 0.14
∘ Mean arterial pressure	87.3 ± 10.9	92.9 ± 7.93	*P* = 0.20
∘ SpO2	97.8 ± 1.5	98.4 ± 1.0	*P* = 0.30
**Post-challenge changes, mean** **±SD**			
∘ Systolic	5.8 ± 12.8	−2.7 ± 10.4	*P* = 0.12
∘ Diastolic	3.0 ± 7.02	−2.0 ± 8.2	*P* = 0.36
∘ Mean arterial pressure	3.9 ± 8.0	−1.0 ± 7.8	*P* = 0.18
∘ SpO2	1.4 ± 1.3	0.50 ± 1.3	*P* = 0.13
**Multidimensional Dyspnea Profile (MDP)**			
Sensation/Response (0–10 scale)			
Unpleasant/discomfort	3.2 ± 2.2	3.7 ± 3.1	*P* = 0.68
Requires effort	3.8 ± 3.1	3.3 ± 3.1	*P* = 0.72
Not getting enough air	2.4 ± 2.2	3.7 ± 2.8	*P* = 0.26
Feel constricted	1.6 ± 1.6	1.6 ± 1.8	*P* = 1.00
Requires mental effort	3.7 ± 3.1	4.3 ± 3.9	*P* = 0.71
Breathing a lot	4.7 ± 3.5	5.4 ± 2.5	*P* = 0.62
Depressed	0.1 ± 0.3	0.6 ± 1.9	*P* = 0.42
Anxious	6.5 ± 8.9	4.2 ± 3.6	*P* = 0.46
Frustrated	1.0 ± 2.2	0.0 ± 0.0	*P* = 0.17
Angry	0.0 ± 0.0	0.0 ± 0.0	–
Afraid	1.5 ± 2.7	2.2 ± 3.7	*P* = 0.63

## Results

A total of 12 persons with epilepsy (PWE) and 10 healthy controls were enrolled in the study and qualified for the fMRI scan. Out of 12 PWE, one patient could not complete the breathing task in the scanner, and one was excluded due to excessive head motion. Therefore, 10 PWE and 10 HCs completed the study and met inclusion/exclusion criteria standards. The mean age among PWE was 35.9 ± 14.2 years, HC was 33.4 ± 7.3, and there was no significant difference in age across groups (*p* = 0.63).

### Demographic and Clinical Characteristics

There was no significant difference in participants' age, sex, BMI, and baseline BP distributions between the groups. In addition, the multidimensional dyspnea questionnaire completed post-scan did not show any significant difference in unpleasant or discomfort experienced by the patients compared to healthy controls (Table 2). A total of 9/10 (90%) PWE were known to have at least one generalized tonic-clonic seizure per year. None were known to have peri-ictal central apnea (refer to the Supplementary Table 1 for PWE characteristics).

**Table 2:** Breathing challenge fMRI results.

	**MNI coordinates**			**Cluster size (K_**E**_)**	***p*-value (cluster-corrected)**	**Z score**
**Region**	**X**	**Y**	**Z**			
**Analysis 1: Breathing challenge: PWE**						
Dorsal raphe nucleus	0	−30	−14	55	P_FWE_ < 0.001	4.43
Locus ceruleus	4	−34	−26	72	P_FWE_ < 0.001	4.92
Lateral hypothalamus	−4	−8	−8	146	P_FWE_ < 0.001	5.07
Rostral pons	0	−26	−28	80	P_FWE_ < 0.001	4.22
Posterior thalamus	8	−16	−4	56	P_FWE_ < 0.001	4.55
Periaqueductal gray	0	−26	−6	55	P_FWE_ = 0.001	3.44
**Analysis 2: Breathing challenge: HC**						
Ventral respiratory group	12	−44	−44	104	P_FWE_ < 0.05	4.10
Substantia nigra	6	−12	−16	26	P_FWE_ < 0.05	3.94
**Analysis 3: PWE vs. HC**						
Dorsal raphe nucleus	0	−32	−16	37	P_FWE_ < 0.05	3.73

### BOLD Activation Patterns

We found significantly greater brainstem BOLD activations in PWE compared to HC during breathing challenges. As shown in Table 1, in the PWE group, we observed a pattern of activation in the raphe nucleus, locus coeruleus, hypothalamus, rostral pons, posterior thalamus, and periaqueductal gray regions (*p* < 0.001 cluster FWE-corrected). For the HC group, consistent activation of the ventral respiratory group and midbrain was observed. Group comparison showed significantly greater activation in the dorsal raphe nucleus among PWE (*p* < 0.05 cluster FWE-corrected) as shown in Figures 1A,B.

### Seed-Based Connectivity Analysis

**Figure 2:**
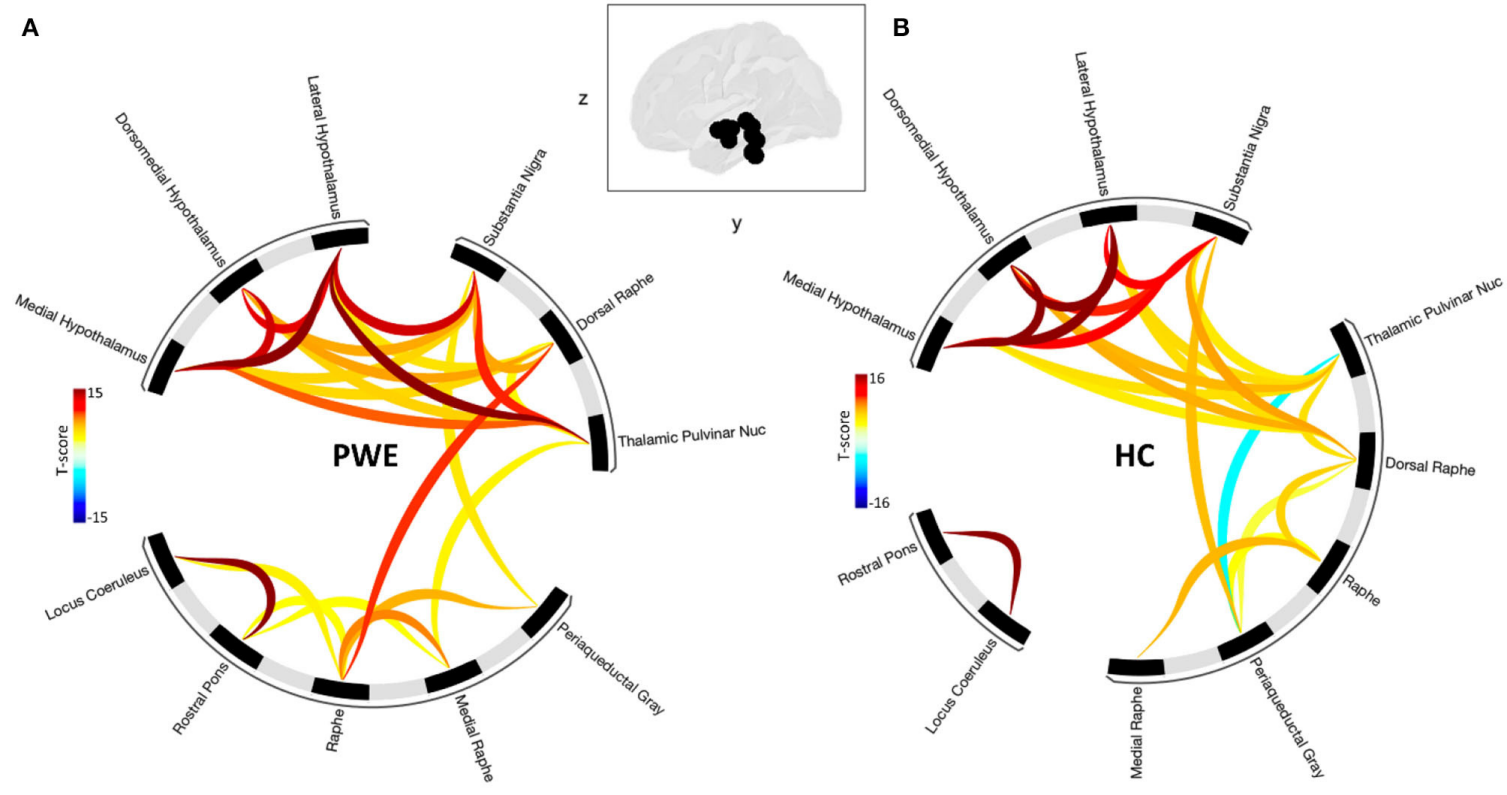
Shows seed-seed functional network connectivity results as cluster-level interference ring. **(A)** The PWE group was characterized by a higher number of connections and higher mean connectivity strength compared to **(B)** healthy controls. Glass brain image represent seed regions used in the analysis. Dark to red connectivity lines signify stronger positive connectivity, lighter to blue connectivity lines denote decreased or negative connectivity. Functional network connectivity cluster threshold: p<0.05 cluster-level p-FDR-corrected (MVPA omnibus test); connection threshold.

Grouped second-level seed-based functional connectivity analyses showed an increase in brain connectivity between regions of the brainstem plus structures during CO_2_ challenge among PWE compared to HCs (Figure 2). PWE had significantly greater seed-seed connectivity and recruited more hypothalamus, pulvinar thalamus to brainstem structures during the breathing challenge compared to HC. This implies that an overall greater effort is required among PWE to complete the same breathing challenge done by HC with little effort. Statistical significance is reported at cluster threshold: *p* < 0.05 cluster level p-FDR-corrected, multivariate statistic parametric (MVPA) omnibus test, and connection threshold. Seed regions in the cluster-level interference ring (Figure 2) are sorted using hierarchical clustering in Conn toolbar.

## Discussion

In this functional magnetic resonance imaging (fMRI) case-control study of breathing responses to hypercapnic challenges, we found BOLD activation of respiratory centers, most significantly in the dorsal raphe nucleus, in PWE compared to HC. In addition, we found pathological functional connectivity alterations in brainstem and adjacent subcortical regions known to specifically modulate breathing as previously implicated in SUDEP(5, 7). Carbon dioxide (CO_2_) is potently vasoactive (12) and clearly produces distinct BOLD alterations, and our case-controlled approach suggests significant activations in regions with chemosensing properties in PWE. Serotonergic [5-hydroxytryptamine (5-HT)] dorsal raphe neurons are central chemoreceptors that project rostrally to modulate arousal (21), are highly sensitive to pH and CO_2_, (22), and are consistent with the observations here.

Brainstem damage is known to occur in PWE (5, 7, 23, 24). Epilepsy is associated with progressive, structural brain changes detectable on MR imaging (25–27), including some which affect cardiorespiratory control sites (24). Volume loss has been seen in key autonomic and breathing control sites (periaqueductal gray, raphe nuclei, and medial posterior thalamus) (5). In support, a series of SUDEP postmortem cases have shown significant alterations in the ventrolateral medulla and in the medullary raphe nucleus, essential regions for human central control of respiration, suggesting compromised respiratory network hubs. Reductions in somatostatin neurons (SST) and neurokinin-1 receptor (NK1R) in the ventrolateral medulla and reduction of 5-HT transporter in the raphe nucleus were seen in brains of patients who died of SUDEP compared to controls (sudden death patients without epilepsy) (7). Altered medullary neuromodulatory systems, including 5-HT and galaninergic systems, indicate that both structural and functional brainstem abnormalities exist in SUDEP patients. Alterations in pre-BotC NK1R and SST neurons have also been reported in other sudden death syndromes (28), an entity with similarities to SUDEP.

There are few human functional imaging studies of respiratory control. To our knowledge, all done in healthy subjects. One fMRI study used CO_2_ inhalation in healthy volunteers; BOLD signal changes were seen in dorsal rostral pons, inferior ventral pons, dorsal and lateral medulla, thalamus, and putamen(10). Another such study of voluntary respiration (hyperpnea) revealed increased BOLD responses in the brainstem (dorsal medulla) (29).

Brainstem dysfunction in respiratory homeostasis is likely underpinned by 5-HT (24, 30). 5-HT is a key chemosensing neurotransmitter that is central to brainstem hypercapnic and arousal mechanisms and strongly implicated in SUDEP (24). Reduced peri-ictal firing of raphe 5-HT neurons during cardiorespiratory dysfunction has been demonstrated in animals (31). Reduced 5-HT can cause post-ictal respiratory arrest and death in SUDEP models, preventable with selective-serotonin reuptake inhibitor (SRI) pretreatment (32, 33). In humans, PWE taking SRIs had shortened peri-ictal apnea (24). Peri-ictal breathing dysfunction includes post-convulsive central apnea and prolonged ictal central apnea, both of which elevate pCO_2_ (8). Altered 5-HT tone has been reported in high-risk patients who suffer such peri-ictal breathing difficulties (34). Impaired CO_2_ chemosensing and failed arousal may underlie SUDEP mechanisms, and premortem identification of abnormal chemosensing using hypercapnic ventilatory responses [HCVR (increase in minute ventilation induced by an increase in end-tidal CO_2_)] has been posited as a potential biomarker(24). HCVR slopes have been shown to be inversely associated with post-ictal hypercapnia duration and magnitude (24). However, large variability in HCVR in PWE (24) and HC (35) poses a challenge for risk stratification that may be overcome by the fMRI approach shown here.

We found fMRI evidence of abnormal chemosensing in the form of a striking pattern of hypercapnia-related BOLD activations in PWE compared to HC, most marked in the dorsal raphe nuclei, a chemosensing region known to be richly innervated by 5-HT neurons. Hypercapnic activation enhances minute ventilation for blood gas stabilization and is critical for respiratory homeostasis (36, 37). The respiratory drive is tightly regulated by CO_2_ concentration, and increased pCO_2_ is a powerful arousal stimulus (38). Both may be relevant to SUDEP. CO_2_-enriched CSF rouses wild-type mice but fails to rouse 5-HT neuron-deficient mice (39). In our study, this abnormally enhanced activation compared to HC suggests abnormal, enhanced recruitment of chemosensing neurons in respiratory hemostasis for restoration of normocarbia, indicating a system under significant “stress.” In contrast, grouped analysis of HC showed less activation, suggesting efficient homeostasis to hypercapnic challenges. Consistent with the BOLD activations described here, we found evidence for abnormal circuitry with increased connectivity between multiple sites with chemosensing properties in PWE compared to HC. Pathologically increased connectivity within what may be called the breathing network may indicate a tendency to impaired recovery from generalized tonic-clonic seizure (GTCS)-induced breathing compromise. Altered networking among autonomic and breathing-related brain areas in patients with a high risk of SUDEP has been described in two previous resting-state fMRI studies (23, 40).

Further, one-sample *t*-test group-level BOLD analysis in PWE showed a number of additional brain regions known to participate in breathing control, including lateral hypothalamus, periaqueductal gray (PAG), posterior thalamus, rostral pons, and locus ceruleus (41–43). Activation of the lateral hypothalamus, a chemosensing region, supports preclinical, within-subject neuroplastic reports of enhanced orexinergic influence on cardiorespiration and autonomic networks prior to SUDEP (44). Volume loss in the periaqueductal gray and medial posterior (pulvinar) thalamus has been described in PWE, and both structures are implicated in respiratory control (5, 45). These network abnormalities may reflect pathomechanisms that increase the risk for breathing dysfunction, particularly in circumstances under which autonomic and respiratory processes are challenged, such as during and after GTCS.

The results of this study suggest distinct differences between PWE and HC. These findings are encouraging and raise the possibility that fMRI biomarkers for patients at high risk of SUDEP may be feasible and are worthy of further study. In particular, stratification of potential BOLD activation extents and locations depending on known presence/absence of GTCS, GTCS frequency, and electroclinical features, such as peri-ictal apnea, O_2_ desaturations, and seizure durations, are the important next steps.

There are several notable limitations here, including small sample size and the lack of correlation between ventilatory parameters with BOLD findings. Thus, there is no stratification of BOLD in PWE into those with or without documented breathing dysfunction since not all patients had epilepsy monitoring unit assessments for observation of habitual peri-ictal breathing compromise. Furthermore, better powered studies designed to examine the correlation of known SUDEP risk factors (GTCS, age at onset, and duration of epilepsy) and electroclinical seizure features, such as prolonged ictal central apnea and post-convulsive central apnea, are necessary for further characterization of these potential premortem SUDEP imaging biomarkers. To maximize brainstem resolution, fMRI was limited to a narrow field of view focus limited to the brainstem. Most brainstem nuclei are in mm resolution, and the imaging protocol of our preliminary study was limited by the resolution of the BOLD MRI sequence; dedicated high resolution EPI sequence is needed to improve signal to noise ratio at the brain stem. Further expansion of this work to include cortical structures with multimodal polygraphy (cardiorespiratory and blood pressure monitoring) during MRI is currently underway.

## Data Availability Statement

The raw data supporting the conclusions of this article will be made available by the authors, without undue reservation.

## Ethics Statement

The studies involving human participants were reviewed and approved by University of Texas Health Sciences Center Houston Institutional Review Board. The patients/participants provided their written informed consent to participate in this study.

## Author Contributions

JoH, NL, and SL contributed to conception and design of the study. MR and JaH organized the study database. JoH performed the imaging, statistical analysis, and wrote the first draft of the manuscript. NL, KS, TS, LL, and SL wrote sections of the manuscript. All authors contributed to manuscript revision, read, and approved the submitted version.

## Funding

This study was supported by grants from the Center for SUDEP Research: NIH/NINDS U01-NS090405, NIH/NINDS U01-NS090407, and NIH/NINDS U01-NS090414.

## Conflict of Interest

The authors declare that the research was conducted in the absence of any commercial or financial relationships that could be construed as a potential conflict of interest.

## Publisher's Note

All claims expressed in this article are solely those of the authors and do not necessarily represent those of their affiliated organizations, or those of the publisher, the editors and the reviewers. Any product that may be evaluated in this article, or claim that may be made by its manufacturer, is not guaranteed or endorsed by the publisher.
