# Supplementary material for: Functional MRI Correlates of Carbon Dioxide Chemosensing in Persons With Epilepsy
Source: Front Neurol. 2022 Jul 7;13:896204. doi: 10.3389/fneur.2022.896204 (PMC9301231; doi:10.3389/fneur.2022.896204)
Supplement: Supplementary file 1 [file Table_1.DOCX]

**Supplementary Table:**

**Patient epilepsy characteristics:**

|  | N (%) | range  (min – max) | Mean ± SD |
| --- | --- | --- | --- |
| Age Of Epilepsy Onset (Years) | 10 | 8 – 44 | 21.40 ± 9.5 |
| Epilepsy Duration (Years) | 10 | 2 – 49 | 14.20 ± 14.8 |
| No. Of Seizures In EMU (N) | 9 | 0 – 16 | 5.89 ± 6.62 |
| Epileptogenic Zone:  Generalized  Bi-Temporal  Temporal  Frontal  Unknown | 1 (10%)  1 (10%)  6 (60%)  1 (10%)  1 (10%) |  |  |
| GTC Seizure Frequency Per Year:  0  1-2  3-12 | 1 (10%)  5 (50%)  4 (40%) |  |  |
| Postictal Central Apnea:  Yes  No  Unknown | 0  8 (80%)  2 (20%) |  |  |
